# Web-based tool for dynamic functional outcome after acute ischemic stroke and comparison with existing models

**DOI:** 10.1186/s12883-014-0214-z

**Published:** 2014-11-25

**Authors:** Ruijun Ji, Wanliang Du, Haipeng Shen, Yuesong Pan, Penglian Wang, Gaifen Liu, Yilong Wang, Hao Li, Xingquan Zhao, Yongjun Wang, On behalf of China National Stroke Registry (CNSR) investigators

**Affiliations:** Tiantan Comprehensive Stroke Center, Beijing Tiantan Hospital, Capital Medical University, No. 6 Tiantanxili, Beijing, 100050 Dongcheng District China; China National Clinical Research Center for Neurological Diseases (NCRC-ND), Beijing, China; Department of Statistics and Operation Research, University of North Carolina, Chapel Hill, NC USA

**Keywords:** Acute ischemic stroke, Prognosis, Risk model

## Abstract

**Background:**

Acute ischemic stroke (AIS) is one of the leading causes of death and adult disability worldwide. In the present study, we aimed to develop a web-based risk model for predicting dynamic functional status at discharge, 3-month, 6-month, and 1-year after acute ischemic stroke (Dynamic Functional Status after Acute Ischemic Stroke, DFS-AIS).

**Methods:**

The DFS-AIS was developed based on the China National Stroke Registry (CNSR), in which eligible patients were randomly divided into derivation (60%) and validation (40%) cohorts. Good functional outcome was defined as modified Rankin Scale (mRS) score ≤ 2 at discharge, 3-month, 6-month, and 1-year after AIS, respectively. Independent predictors of each outcome measure were obtained using multivariable logistic regression. The area under the receiver operating characteristic curve (AUROC) and plot of observed and predicted risk were used to assess model discrimination and calibration.

**Results:**

A total of 12,026 patients were included and the median age was 67 (interquartile range: 57–75). The proportion of patients with good functional outcome at discharge, 3-month, 6-month, and 1-year after AIS was 67.9%, 66.5%, 66.9% and 66.9%, respectively. Age, gender, medical history of diabetes mellitus, stroke or transient ischemic attack, current smoking and atrial fibrillation, pre-stroke dependence, pre-stroke statins using, admission National Institutes of Health Stroke Scale score, admission blood glucose were identified as independent predictors of functional outcome at different time points after AIS. The DFS-AIS was developed from sets of predictors of mRS ≤ 2 at different time points following AIS. The DFS-AIS demonstrated good discrimination in the derivation and validation cohorts (AUROC range: 0.837-0.845). Plots of observed versus predicted likelihood showed excellent calibration in the derivation and validation cohorts (all r = 0.99, P < 0.001). When compared to 8 existing models, the DFS-AIS showed significantly better discrimination for good functional outcome and mortality at discharge, 3-month, 6-month, and 1-year after AIS (all P < 0.0001).

**Conclusion:**

The DFS-AIS is a valid risk model to predict functional outcome at discharge, 3-month, 6-month, and 1-year after AIS.

**Electronic supplementary material:**

The online version of this article (doi:10.1186/s12883-014-0214-z) contains supplementary material, which is available to authorized users.

## Background

Stroke is one of the leading causes of death and adult disability worldwide and acute ischemic stroke (AIS) accounts for about 85% of all stroke cases [1,2]. At time of admission, clinicians are frequently requested to estimate the likelihood of good functional outcome in post-stroke period. During the past decades, a number of prognostic models for AIS have been developed [3-18]. Except the IScore and ASTRAL score [15,17], majority of them have not been externally validated. Meanwhile, none of them is recommended by international stroke guidelines and widely used in routine clinical practice or clinical trials [3,19]. In addition, the majority of existing models are designed to provide prognostic information for a single time point after AIS, such as at discharge [13], 30-day [10], 3-month [4-7,9,17], 6-month [14], 9-month [16] or 1-year [8,11] after onset. Although few recently proposed risk models could provide dynamic prognostic information at multiple time points after AIS [15,18], they mainly focused on mortality instead of functional status. In real-world practice, patients, family members, clinicians and researchers usually concern about not only the likelihood of survival, but also the likelihood of survival with function recovery after AIS.

In the study, we aimed to develop and validate a risk model to provide dynamic prognostic information on functional status at multiple time points (discharge, 3-month, 6-month, and 1-year) after AIS (Dynamic Functional Status after Acute Ischemic Stroke, DFS-AIS). Furthermore, we compared discrimination of the DFS-AIS and existing AIS models with regard to both functional outcome and mortality at discharge, 3-month, 6-month, and 1-year after AIS.

## Methods

### Study population

The derivation and validation cohort originated from the largest stroke registry in China, the China National Stroke Registry (CNSR) [20], which was a nationwide, multicenter, and prospective registry of consecutive patients with acute cerebrovascular events from September 2007 to August 2008. Briefly, hospitals in China are classified into 3 Levels: I (community hospitals); II (hospitals that serve several communities); and III (central hospitals for a certain district or city). Level III hospital is usually an academic center and more medically advanced than level I and II hospitals. Totally, 242 potential sites including 114 Level III, 71 Level II, and 57 Level I hospitals, from both urban and rural area, were initially identified by soliciting application. The CNSR steering committee evaluated research capability and commitment to the registry of each hospital with preliminary survey. Finally, a total of 132 hospitals including 100 Level III and 32 Level II were selected, which cover 27 provinces and 4 municipalities across China. Trained research coordinators at each institute reviewed medical records daily to identify, consent and enroll consecutively eligible patients. To be eligible for this study, subjects had to meet the following criteria: (1) age 18 years or older; (2) hospitalized with a primary diagnosis of AIS according to the World Health Organization criteria [21]; (3) stroke confirmed by head computerized tomography (CT) or brain magnetic resonance imaging (MRI); (4) direct admission to hospital from a physician’s clinic or emergency department; (5) written informed consent from patients or their legal representatives. For the present study, patients who received thrombolysis (n = 389, 3.1%) (Additional file 1: Table S1) were excluded since thrombolysis alters the natural history of disability after stroke [22]. However, they were included in sensitivity analysis. The scientific use of data registered in the CNSR was approved by the central institutional review board at Beijing Tiantan hospital and local ethical committees at each participating hospital (Additional file 1).

### Data collection and variables definition

Standardized case report form (CRF) was used for data collection in the CNSR network. The relevant data was extracted from the medical records by trained research coordinators. Data from each CRF were manually checked for completeness, correct coding, and proper application of diagnostic algorithm by a research specialist from an independent contract research organization. In the study, the following candidate variables were analyzed: (1) demographics (age and gender); (2) stroke risk factors: hypertension (history of hypertension or anti-hypertensive medication use), diabetes mellitus (history of diabetes mellitus or anti-diabetic medication use), dyslipidemia (history of dyslipidemia or lipid-lowering medication use), atrial fibrillation (history of atrial fibrillation or documentation of atrial fibrillation on admission), coronary heart disease, history of stroke/TIA, current smoking, and excess alcohol consumption (≥2 standard alcohol beverages per day); (3) pre-existing comorbidities: congestive heart failure, valvular heart disease, peripheral artery disease, chronic obstructive pulmonary disease (COPD), hepatic cirrhosis, peptic ulcer or previous gastrointestinal bleeding (GIB), renal failure, Alzheimer’s disease/dementia, and cancer; (4) pre-stroke dependence (modified Rankin Scale score ≥ 3); (5) pre-admission antithrombotic medications: anticoagulant with warfarin (for atrial fibrillation) or anti-platelet medication (aspirin, clopidogrel, or extended release dipyridamole combined with aspirin); (6) pre-admission statins using; (7) transportation mode to hospital (dichotomized as by emergency medical system [EMS] or private transportation [such as by taxi or private car]); (8) Time from onset to hospital arrival (hours); (9) admission systolic and diastolic blood pressure (mmHg); (10) admission stroke severity based on National Institutes of Health Stroke Scale (NIHSS) score; (11) stroke subtypes according to the Oxfordshire Community Stroke Project (OCSP) criteria [23], where AIS was classified into partial anterior circulation infarct (PACI), total anterior circulation infarct (TACI), lacunar infarction (LACI), and posterior circulation infarct (POCI). We did not use TOAST subtype because it requires the results of investigations that usually are not available at the time of admission for many patients; (12) admission blood glucose (mmol/L).

### Functional outcome assessment

The modified Rankin Scale (mRS) was used to assess functional outcome at different time points (discharge, 3-month, 6-month and 1-year) after AIS. Treating physician evaluated patients’ functional status at discharge. A central follow-up for functional status at 3-month, 6-month and 1-year after onset was made by telephone interview by trained interviewers based on a standardized interview protocol. Good functional outcome was defined as mRS ≤ 2 at discharge, 3-month, 6-month, and 1-year after AIS, respectively.

### Statistical analysis

We aimed to derivate and validate a risk model to predict functional outcome at multiple time points (discharge, 3-month, 6-month, and 1-year) after AIS by using patient characteristics commonly available at presentation. In order to guarantee the accuracy of prediction, we separately developed 4 prognostic rules for each outcome measures. For the practicability of the model, we developed a web-based and user-friendly calculator, which can automatically provide prognostic information at different time points after AIS.

The eligible patients (an additional figure shows this in more detail [see Additional file 1: Figure S1]) in the CNSR were randomly divided into derivation (60%) and validation (40%) cohorts. Model building was performed exclusively in the derivation cohort (n = 7,215). In univariate analysis, Chi-square or Mann–Whitney test was used as appropriate. Multivariate logistic regression was used to determine independent predictors of good functional outcome (mRS ≤ 2) at discharge, 3-month, 6-month and 1-year after AIS, respectively. Candidate variables were those with biologically plausible link to good functional outcome after AIS on the basis of prior publication and those associated with good functional outcome after AIS on univariate analysis (P ≤ 0.1). On multivariate analysis, backward stepwise method was used. To test for collinearity between covariates of the final multivariable model, the tolerance and variance inflation factor (VIF) of each covariate were calculated. The resulting DFS-AIS was then validated by assessing model discrimination and calibration in the validation cohort (n = 4,811) [24]. Discrimination was assessed by calculating the area under the receiver operating characteristic curve (AUROC). Calibration was assessed by performing the Hosmer-Lemeshow goodness-of-fit test. Due to that the Hosmer-Lemeshow test has been shown to be overly sensitive to trivial deviations from the ideal fit when the sample size is large [25], model calibration was also graphically depicted in the plot of observed versus predicted risk according to 10 deciles of predicted risk.

Furthermore, we compared discrimination of the DFS-AIS and existing AIS models with regard to both functional outcome (mRS ≤ 2) and mortality at discharge, 3-month, 6-month and 1-year after AIS in the overall cohort (n = 12,026). The primary criterion for selection of compared model was whether all elements required for the model were available in our dataset. Meanwhile, due to that a systematical review on prognostic models of acute stroke published in 2001 indicated that none of the existing prognostic models have been sufficiently well developed and validated to be useful in either clinical practice or research, we included only AIS risk models developed after 2001. Finally, 8 risk models (the Weimar’s survival and functional model [9], König’s survival and functional model [12], GWTG score [13], IScore [15], ASTRAL score [17], and PLAN score [18]) were selected. Discrimination of these models (AUROC) was compared using the test proposed by Delong et al. [26]. Sensitivity, specificity, positive predict value (PPV), and negative predictive value (NPV) was calculated at each model’s maximum Youden Index. Missing data were coded as following: 25 patients (0.2%) with missing data on medical history of current smoking, 110 (0.9%) of excess alcohol consumption, 30 (0.2%) of Alzheimer disease/dementia, 50 (0.4%) of hepatic cirrhosis were coded as absence. Sixty-sever patients (0.6%) with missing or unknown time from stroke onset to hospital arrival were coded as median.

All tests were 2-tailed and statistical significance was determined at α level of 0.05. Regarding the number of comparisons done in comparing discrimination of prognostic models, we use more restricted criterion (0.001) to declare significant difference. Statistical analysis was performed using SAS 9.3 (SAS Institute, Cary, NC), SPSS 20.0 (SPSS Inc., Chicago, IL), and Medcalc software 12.3 (MedCalc, Ostend, Belgium).

## Results

### Patient characteristics

Patient characteristics of the derivation and validation cohorts are shown in Table 1. From September 2007 to August 2008, a total of 12,026 patients in the CNSR were eligible for the study and included in final analysis (an additional figure shows this in more detail [see Additional file 1: Figure S1]). The median age was 67 (IQR 57–75) and 61.6% were male. The median length of hospital stay was 14 days (IQR: 10–20). The median admission NIHSS score was 5 (IQR: 2–9). The proportion of good functional outcome (mRS ≤ 2) at discharge, 3-month, 6-month, and 1-year after AIS was 67.9%, 66.5%, 66.9% and 66.9%, respectively. The eligible patients were randomly divided into derivation (60%, n = 7,215) and validation cohort (40%, n = 4,811), which were matched with respect to baseline characteristics and overall rate of good functional outcome (mRS ≤ 2) at different time points following AIS (Table 1).

**Table 1:** **Patient characteristics**

	**Overall cohort (N = 12026)**	**Derivation cohort (N = 7215)**	**Validation cohort (N = 4811)**	**P value**
Demographics				
Age, y, median (IQR)	67 (57–75)	67 (57–75)	67 (57–75)	0.35
Gender (male), n (%)	7411 (61.6)	4461 (61.8)	2950 (61.3)	0.58
Stroke risk factors, n (%)				
Hypertension	7703 (64.1)	4602 (63.8)	3101 (64.5)	0.46
Diabetes mellitus	2615 (21.7)	1580 (21.9)	1035 (21.9)	0.62
Dyslipidemia	1349 (11.2)	797 (11.0)	552 (11.5)	0.47
Atrial fibrillation	870 (7.2)	543 (7.5)	327 (6.8)	0.13
Coronary artery disease	1714 (14.3)	1063 (14.7)	651 (13.5)	0.07
History of stroke/TIA	4113 (33.6)	2403 (33.3)	1635 (34.0)	0.44
Current Smoking	4750 (39.5)	2846 (39.4)	1904 (39.6)	0.89
Excess alcohol consumption	1844 (15.3)	1124 (15.6)	720 (15.0)	0.37
Pre-existing comorbidities, n (%)				
Congestive heart failure	239 (2.0)	151 (2.1)	88 (1.8)	0.32
Valvular heart disease	284 (2.4)	171 (2.4)	113 (2.3)	0.95
COPD	138 (1.1)	80 (1.1)	58 (1.2)	0.66
Hepatic cirrhosis	42 (0.4)	24 (0.3)	18 (0.4)	0.76
Peptic ulcer or previous GIB	411 (3.4)	235 (3.3)	178 (3.7)	0.24
Alzheimer’s disease/Dementia	166 (1.4)	95 (1.3)	71 (1.5)	0.47
Cancer	222 (1.8)	140 (1.9)	82 (1.7)	0.36
Pre-stroke dependence (mRS ≥ 3), n (%)	1140 (9.5)	684 (9.5)	456 (9.5)	0.99
Pre-admission antithrombotic therapy, n (%)	2246 (18.7)	1345 (18.6)	901 (18.7)	0.91
Warfarin (for atrial fibrillation)	272 (2.3)	170 (2.4)	102 (2.1)	
Antiplatelet using	2208 (16.7)	1201 (16.6)	807 (16.8)	
Pre-admission statins using, n (%)	5175 (43.0)	3066 (42.5)	2109 (43.8)	0.15
Transport to hospital by EMS, n (%)	1826 (15.2)	1075 (14.9)	751 (15.6)	0.29
Time from onset to arrival (hours), median (IQR)	24 (7–64)	24 (7–64)	24 (7–65)	0.83
Admission SBP (mm Hg), median (IQR)	150 (136–164)	150 (135–163)	150 (136–164)	0.76
Admission DBP (mm Hg), median (IQR)	89 (80–96)	89 (80–96)	89 (80–96)	0.67
Admission NIHSS score, median (IQR)	5 (2–9)	5 (2–10)	5 (2–9)	0.33
NIHSS = 0-4	6152 (51.2)	3686 (51.1)	2466 (51.3)	0.31
NIHSS = 5-9	3159 (26.3)	1873 (26.0)	1286 (26.7)	
NIHSS = 10-14	1312 (10.9)	783 (10.9)	529 (11.0)	
NIHSS ≥ 15	1403 (11.7)	873 (12.1)	530 (11.0)	
OCSP subtypes, n (%)				0.69
Partial anterior circulation infarct (PACI)	6698 (55.7)	4025 (55.8)	2673 (55.6)	
Total anterior circulation infarct (TACI)	1035 (8.6)	623 (8.6)	412 (8.6)	
Lacunar infarction (LACI)	2252 (18.7)	1365 (18.9)	887 (18.4)	
Posterior circulation infarct (POCI)	2009 (17.1)	1180 (16.8)	829 (17.6)	
Admission blood glucose (mmol/L), median (IQR)	6.2 (5.5-7.0)	6.2 (5.5-7.0)	6.2 (5.5-7.1)	0.30
Length of hospital stay, median (IQR)	14 (10–20)	14 (10–20)	14 (11–20)	0.55
mRS ≤ 2 within 1 year after onset, n (%)				
At discharge	8160 (67.9)	4885 (67.7)	3275 (68.1)	0.68
At 3-month	7994 (66.5)	4771 (66.1)	3223 (67.0)	0.33
At 6-month	8050 (66.9)	4806 (66.6)	3244 (67.4)	0.35
At 12-mont	8047 (66.9)	4817 (66.8)	3230 (67.1)	0.68
Mortality within 1 year after onset, n (%)				
At discharge	468 (3.9)	276 (3.8)	192 (4.0)	0.67
At 3-month	990 (8.2)	606 (8.4)	384 (8.0)	0.44
At 6-month	1270 (10.6)	774 (10.7)	496 (10.3)	0.49
At 12-mont	1602 (13.2)	995 (13.8)	607 (12.6)	0.07

*Abbreviation:* *IQR* Interquartile Range, *TIA* Transient Ischemic Attack, *COPD* Chronic obstructive pulmonary disease, *mRS* Modified Rankin Scale, *EMS* Emergency Medical System, *SBP* Systolic Blood Pressure, *DBP* Diastolic Blood Pressure, *NIHSS* National Institutes of Health Stroke Scale score, *OCSP* Oxfordshire Community Stroke Project.

### Derivation of the DFS-AIS

**Figure 1:**
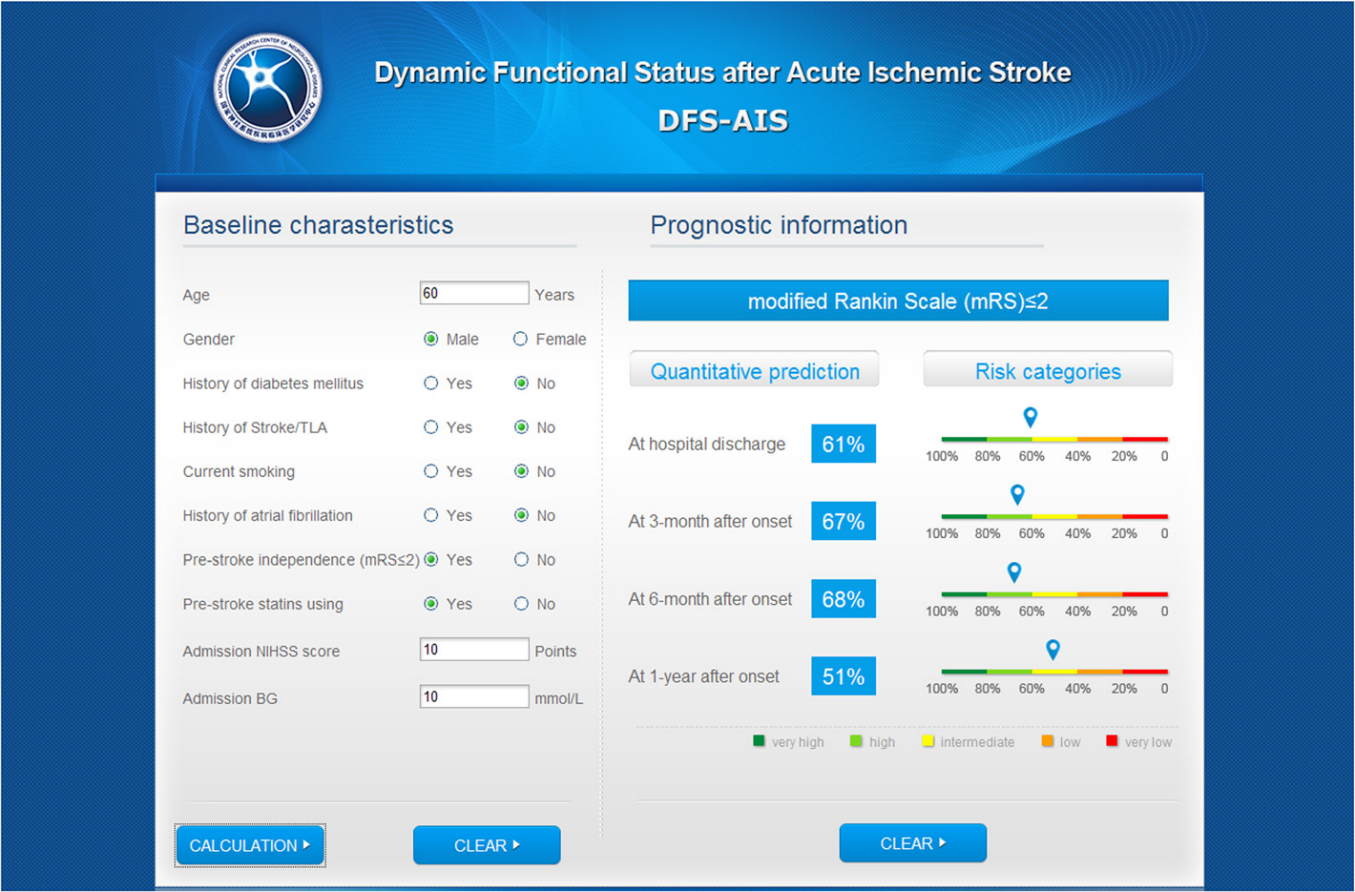
**The web-based calculator for the DFS-AIS.**

The multivariate analysis for predictors of good functional outcome (mRS ≤ 2) at discharge, 3-month, 6-month, and 1-year after AIS is shown in Table 2. Age, gender, medical history of diabetes mellitus, stroke/TIA (for mRS ≤ 2 at 3-month, 6-month and 1-year after onset), current smoking (for mRS ≤ 2 at discharge) and atrial fibrillation (for mRS ≤ 2 at 3-month, 6-month and 1-year after onset), pre-stroke dependence, pre-stroke statins using (for mRS ≤ 2 at 3-month, 6-month and 1-year after onset), admission NIHSS score, admission blood glucose were identified as independent predictors of functional outcome at different time points after AIS. The tolerance of covariates in the final multivariable model ranged 0.80-0.99 and the VIF 1.01-1.21 (Table 2). The probability of good functional outcome (mRS ≤ 2) at discharge, 3-month, 6-month, and 1-year after AIS can be estimated for an individual patient by a web-based calculator (Figure 1) (www.dfs-ais.com).

**Table 2:** **Multivariable analysis**

**Predictors**	**Discharge mRS** ≤ 2*			**3-month mRS** ≤ 2*			**6-month mRS** ≤ 2*			**1-year mRS** ≤ 2*		
	**ß-coefficient**	**OR (95% C.I.)**	**P**	**ß-coefficient**	**OR (95% C.I.)**	**P**	**ß-coefficient**	**OR (95% C.I.)**	**P**	**ß-coefficient**	**OR (95% C.I.)**	**P**
Intercept	2.855			4.010			4.355			3.892		
Age (per 1 year increase)	−0.02	0.98 (0.97-0.98)	<0.0001	−0.043	0.96 (0.95-0.96)	<0.0001	−0.050	0.95 (0.95-0.96)	<0.0001	−0.058	0.94 (0.94-0.95)	<0.0001
Gender (male)	0.295	1.34 (1.19-1.52)	<0.0001	0.234	1.26 (1.14-1.40)	<0.0001	0.189	1.21 (1.09-1.34)	<0.0001	0.274	1.32 (1.18-1.47)	<0.0001
Risk factors												
Diabetes mellitus (No)	0.204	1.23 (1.08-1.39)	0.001	0.289	1.34 (1.17-1.52)	<0.0001	0.296	1.34 (1.18-1.53)	<0.0001	0.239	1.27 (1.11-1.45)	<0.0001
Stroke/TIA (No)	…	…	…	0.278	1.32 (1.18-1.49)	<0.0001	0.316	1.37 (1.22-1.55)	<0.0001	0.371	1.45 (1.30-1.62)	<0.0001
Current smoking (No)	0.157	1.17 (1.03-1.33)	0.01	…	…	…	…	…	…	…	…	…
Atrial fibrillation (No)	…	…	…	0.218	1.24 (1.02-1.52)	0.03	0.230	1.26 (1.03-1.53)	0.03	0.303	1.35 (1.11-1.65)	0.003
Pre-stroke independence (mRS ≤ 2)	0.549	1.73 (1.47-2.04)	<0.0001	0.588	1.80 (1.51-2.15)	<0.0001	0.672	1.96 (1.65-2.33)	<0.0001	0.634	1.89 (1.58-2.22)	<0.0001
pre-stroke statins using (Yes)	…	…	…	0.198	1.22 (1.10-1.35)	<0.0001	0.213	1.24 (1.12-1.37)	<0.0001	0.263	1.30 (1.17-1.44)	<0.0001
Admission NIHSS score (per 1 increase)	−0.197	0.82 (0.81-0.83)	<0.0001	−0.211	0.81(0.80-0.82)	<0.0001	−0.196	0.82 (0.81-0.83)	<0.0001	−0.185	0.83 (0.82-0.84)	<0.0001
Admission BG (per 1 mmol/L increase)	−0.044	0.96 (0.94-0.98)	<0.0001	−0.043	0.95 (0.93-0.97)	<0.0001	−0.054	0.95 (0.93-0.97)	<0.0001	−0.061	0.94 (0.92-0.96)	<0.0001

Multivariable logistic regression adjusted for age, gender, stroke risk factors, pre-existing comorbidities, pre-stroke dependence, pre-admission medications (anticoagulant with warfarin, anti-platelet medication, and statins), transport model to hospital, time from onset to hospital arrival, admission systolic and diastolic blood pressure, NIHSS score, OCSP subtypes, and admission blood glucose.

*Probability of good functional outcome (mRS ≤ 2) is calculated by P = e^Y^/(1 + e^Y^), where Y = a + b_1_X_1_ + b_2_X_2_ +∴.. + b_i_X_i_;

*Abbreviation*: *OR* Odds Ratio, *C.I.* Confidence Interval, *VIF* Variance Inflation Factor, *TIA* Transient Ischemic Attack, *mRS* Modified Rankin Scale, *NIHSS* National Institutes of Health Stroke Scale score, *BG* Blood glucose.

### Validation of the DFS-AIS

Discrimination of the DFS-AIS with regard to good functional outcome (mRS ≤ 2) at discharge, 3-month, 6-month, and 1-year after AIS is shown in Additional file 1: Figure S2 (an additional figure shows this in more detail [see Additional file 1: Figure S2]). Similar good discrimination was found in both the derivation (AUROC range: 0.842-0.845) and validation cohort (AUROC range: 0.837-0.841). The Hosmer-Lemeshow tests of the DFS-AIS for good functional outcome at discharge, 3-month, 6-month, and 1-year after AIS were significant in the validation cohort (all P < 0.001). However, a graph of observed versus predicted likelihood of good functional outcome at discharge, 3-month, 6-month, and 1-year after AIS showed a close correlation between observed and predicted risk in both the derivation and validation cohorts (all r = 0.99, P < 0.001) (an additional figure shows this in more detail [see Additional file 1: Figure S3]), which indicated excellent calibration.

### Sensitivity analysis

We completed pre-specified subgroup analyses by age, gender, time intervals from stroke onset to arrival, OCSP subtype and thrombolysis. Similar good discrimination was seen in these subgroups (AUROC range: 0.773-0.917), especially for those patients with TACI subtype (AUROC range: 0.855-0.917) (Table 3).

**Table 3:** **Sensitivity analysis of the DFS-AIS in the overall cohort (n = 12,026)**

	**Discharge mRS** ≤ **2**		**3-month mRS** ≤ **2**		**6-month mRS **≤ **2**		**1-year mRS** ≤ **2**	
	**AUROC**	**95% C.I.**	**AUROC**	**95% C.I.**	**AUROC**	**95% C.I.**	**AUROC**	**95% C.I.**
Age								
≤59	0.845	0.829-0.861	0.826	0.808-0.844	0.820	0.801-0.839	0.813	0.793-0.833
≥60	0.830	0.820-0.839	0.836	0.826-0.846	0.830	0.820-0.840	0.827	0.817-0.837
Gender								
Male	0.834	0.823-0.845	0.832	0.821-0.843	0.834	0.823-0.845	0.834	0.823-0.845
Female	0.843	0.830-0.856	0.849	0.837-0.861	0.846	0.834-0.858	0.845	0.833-0.857
Time from onset to arrival (hours)								
≤3	0.829	0.808-0.850	0.846	0.825-0.867	0.847	0.826-0.868	0.857	0.837-0.877
3-6	0.838	0.808-0.868	0.826	0.796-0.856	0.836	0.807-0.865	0.834	0.805-0.863
6-24	0.834	0.818-0.850	0.845	0.830-0.860	0.842	0.827-0.857	0.848	0.833-0.862
≥24	0.845	0.833-0.856	0.841	0.829-0.853	0.839	0.828-0.850	0.831	0.819-0.842
OCSP subtype								
Lacunar infarction (LACI)	0.809	0.785-0.833	0.819	0.796-0.842	0.829	0.807-0.851	0.816	0.794-0.838
Partial anterior circulation infarct (PACI)	0.827	0.816-0.838	0.821	0.810-0.832	0.822	0.811-0.833	0.826	0.815-0.837
Total anterior circulation infarct (TACI)	0.855	0.830-0.880	0.917	0.900-0.936	0.912	0.892-0.932	0.905	0.885-0.925
Posterior circulation infarct (POCI)	0.853	0.833-0.873	0.846	0.826-0.866	0.840	0.820-0.860	0.845	0.826-0.846
Intravenous or intra-arterial thrombolysis	0.773	0.724-0.822	0.785	0.735-0.833	0.779	0.733-0.825	0.781	0.730-0.832

*Abbreviation:* *mRS* Modified Rankin Scale, *AUROC* Area Under the Receiver Operating Characteristic Curve, *C.I.* Confidence Interval, *OCSP* Oxfordshire Community Stroke Project.

### Comparison of AIS risk models

**Figure 2:**
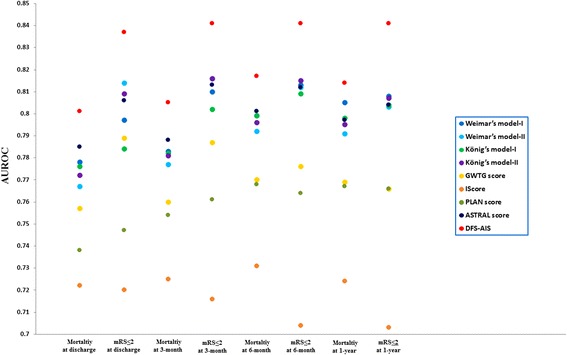
**Comparative evaluation of the DFS-AIS and 8 existing AIS models.** Figure 2 showed discrimination of the DFS-AIS and 8 existing AIS models with regard to good functional outcome (mRS ≤ 2) and mortality at discharge, 3-month, 6-month, and 1-year after AIS in the validation cohort (n = 4,811). The DFS-AIS consistently showed significant better discrimination than compared models with regard to both good functional outcome (mRS ≤ 2) and mortality at discharge, 3-month, 6-month, and 1-year after AIS (all P < 0.0001).

Discrimination of the DFS-AIS and 8 existing AIS models for good functional outcome (mRS ≤ 2) and mortality at discharge, 3-month, 6-month and 1-year after AIS in the validation cohort (n = 4,811) is shown in Figure 2 (additional tables show this in more detail [see Additional file 1: Table S2 and S3]). For good functional outcome prediction, although all tested models showed acceptable discrimination (AUROC ≥ 0.70), the DFS-AIS demonstrated significantly higher AUROC than other models (all P < 0.0001). Meanwhile, the DFS-AIS had the highest maximum Youden Index and associated sensitivity, specificity, PPV and NPV (an additional table shows this in more detail [see Additional file 1: Table S2]). Similar results were found for predicting mortality at discharge, 3-month, 6-month, and 1-year after AIS (Figure 2 and Additional file 1: Table S3). When the comparison was performed in the overall cohort (n = 12,026), similar results was found (see Additional file 1: Table S4 and S5).

## Discussion

In the study, we developed and validated a risk model to predict functional outcome at multiple time points (discharge, 3-month, 6-month, and 1-year) after AIS by using information routinely available at presentation. A web-based risk model (the DFS-AIS) was developed based on the set of independent predictors of each outcome measures and showed good discrimination and calibration in large derivation and validation cohorts. When compared to 8 existing models, the DFS-AIS showed significantly better discrimination with regard to both functional outcome and mortality at discharge, 3-month, 6-month, and 1-year after AIS.

In order to preserve clinical utility of the model for decision-making during acute hospitalization and postdischarge, we used only patient characteristics available at presentation. We chose not to include variables related to in-hospital and postdischarge management, such as intravenous recombinant tissue plasminogen activator (rt-PA) [22], rehabilitation, and persistence of evidence-based secondary prevention medications [27-29], despite the fact that these therapies could influence functional status following AIS. This model therefor predicts the expected functional status following AIS at presentation.

For a clinical prognostic rule to become effective and widely used, it must be reliable, accurate and practicable [3]. For reliability, the DFS-AIS was developed based on large derivation and validation cohorts, which included consecutive patients of AIS from a nationwide stroke registry, was outside of clinical trials, and was more reflective of real-world clinical practice; In addition, by sensitivity analysis, the DFS-AIS demonstrated to be effective for patients with various clinical characteristics, such as different age, gender, time intervals from stroke onset to hospital arrival, and OCSP subtype. For accuracy, the DFS-AIS was proven to be accurate in risk-stratification and outcome prediction for both functional outcome and mortality at multiple time points after AIS. For practicability, the DFS-AIS consists of factors that are readily available at hospital presentation. In addition, by a web-based and user-friendly calculator, users could obtain prognostic information at multiple time points following AIS without doing complex calculation.

To the best of our knowledge, we are the first to systematically compare AIS risk models in a single population. During the past decades, a multitude of AIS predictive models have been developed. Although each of these prognostic rules has inherent strengths and limitations, none of them is widely accepted and used in routine clinical practice or clinical trials [3,19]. With many AIS prognostic rules available, identification of the most accurate and reliable model(s) would be of great value to patients, family members, clinicians, and researchers. In this study, we found that the DFS-AIS consistently demonstrated significant better discrimination than 8 compared models for both functional outcome and mortality at discharge, 3-month, 6-month, and 1-year after AIS (Figure 2, Table S1, and S2). Though promising, one should exercise caution when interpreting the results: first, the study populations for derivation and validation of these AIS risk models are different. The clinical characteristics of our study population were different from those of western cohorts used to develop compared models [13,15,17,18], such as with younger age of AIS onset, higher proportion of male gender, less severity of neurological deficit on admission, and lower rate of mortality at different time points following AIS. It is not our primary aim to compare difference of these cohorts and it is hard to explain the potential reasons due to sorts of difference in study design and population. Second, there might be complex genetic, social, culture, economic factors as well as regional management philosophies and preferences that are difficult to account for when prognostic models are developed or applied to a distinct population. Finally, the intended outcome (functional outcome vs. mortality), outcome assessment methods (Barthel Index vs. mRS), and timing of follow-up (in-hospital vs. 30-day vs. 3-month vs. 6-month vs. 1-year after AIS) are different for these AIS models. One might argue it is not fair to directly compare these models for functional outcome and mortality at various time points after AIS as that they were not designed in that way. However, our intention was not to show limitation of these models beyond the context under which they are developed, but to provide evidence on which model is more appropriate for what kind of outcome (functional outcome vs. mortality) and at what specific time points (discharge, 3-month, 6-month, and 1-year) after AIS.

By offering prediction of functional outcome at multiple time points following AIS, rather than only mortality, the DFS-AIS can be useful for patients, family members, clinicians and researchers, whose primary concern is not the likelihood of survival, but rather the likelihood of survival with recovery of function. This is also important since different clinical care contexts or clinical research may focus on short-term or long-term functional outcome after AIS differently. Meanwhile, the DFS-AIS could be used to monitor the performance of hospitals or stroke units in stroke care. Those hospitals or stroke units which deviate in outcome from the expected outcome (e.g. worse outcome) need to be monitored in detail to identify the potential targets for improvement. In addition, like other prognostic tools, the DFS-AIS may help in randomized clinical trials to stratify study population, in nonrandomized clinical trials to control for case-mix variation, and in controlled clinical trials to select suitable patients and to reduce required sample size [19,30,31].

Our study has limitations that deserve comment. First, like all observational studies, we cannot rule out the possibility that additional variable (unmeasured confounders) might have some impact on functional status after stroke, such as intravenous rt-PA, neuroimaging characteristics, rehabilitation, and persistence of evidence-based secondary prevention medications. However, given our emphasis on early prediction using information at presentation to predict dynamic functional outcome after AIS, the DFS-AIS might be helpful to guide subsequent in-hospital and post-discharge management. Second, our study included only hospitalized stroke patients and those patients died in emergency department or treated in outpatient clinics were not included. Meanwhile, like most registries, our registry required informed consent and selection bias was inevitable [32]. Third, due to that the Medical Research Council score, volume of ischemia on diffusion-weighted imaging, Charlson comorbidity index, Consortium for the Investigation of Vascular Impairment of Cognition scale score, body temperature, and oxygen administration were not routinely collected in the CNSR, several other AIS models cannot be externally validated in the study [4-6,8,11,14,16]. Finally, the DFS-AIS is not externally validated in the study. Meanwhile, both the derivation and validation cohorts originated from Asian population, and therefore, the score needed to be further validated in non-Asian populations.

## Conclusion

The DFS-AIS is a valid clinical model for predicting functional outcome at discharge, 3-month, 6-month, and 1-year after AIS. For clinical practice, the DFS-AIS can be useful for patients, family members, and clinicians by offering prediction of functional outcome at multiple time points following AIS. For clinical research, the DFS-AIS may help in randomized clinical trials to stratify study population, in nonrandomized clinical trials to control for case-mix variation, and in controlled clinical trials to select suitable patients and to reduce required sample size. Further validation of the DFS-AIS in different populations is required.

## Additional file

Additional 1: Table S1. Patient characteristics; **Table S2.** Discrimination of the DFS-AIS and 8 existing models for functional outcome after AIS in the validation cohort (n = 4,811); **Table S3.** Discrimination of the DFS-AIS and 8 existing models for mortality after AIS in the validation cohort (n = 4,811); **Table S4.** Discrimination of the DFS-AIS and 8 existing models for functional outcome after AIS in the overall cohort (n = 12,026); **Table S5.** showed comparison of discrimination of the DFS-AIS and 8 existing models for functional outcome at discharge, 3-month, 6-month and 1-year after AIS in the overall cohort. **Table S6.** showed comparison of discrimination of the DFS-AIS and 8 existing models for mortality at discharge, 3-month, 6-month and 1-year after AIS in the overall cohort. **Figure S1.** Patient flowchart; **Figure S2.** Discrimination of the DFS-AIS for dynamic functional outcome after AIS. **Figure S3.** Plot of observed versus predicted likelihood of good functional outcome at multiple time points after AIS; Additional file 1: The CNSR investigators; Additional file 1: Institutional Review Board within the CNSR network.

## Abbreviations

AIS: Acute ischemic stroke
AUROC: Area under the receiver operating characteristic curve
C.I.: Confidence interval
CNSR: China national stroke registry
COPD: Chronic obstructive pulmonary disease
CRF: Case report form
CT: Computerized tomography
DBP: Diastolic blood pressure
DFS-AIS: Dynamic functional status after acute ischemic stroke
EMS: Emergency medical system
GIB: Gastrointestinal bleeding
IQR: Interquartile range
LACI: Lacunar infarction
MRI: Magnetic resonance imaging
mRS: Modified rankin scale
NIHSS: National Institutes of health stroke scale score
NPV: Negative predictive value
OCSP: Oxfordshire community stroke project
OR: Odds ratio
PACI: Partial anterior circulation infarct
POCI: Posterior circulation infarct
PPV: Positive predict value
SBP: Systolic blood pressure
SE: Standard error
TACI: Total anterior circulation infarct
TIA: Transient ischemic attack
VIF: Inflation factor
WBC: White cell count
