# Supplementary material for: Web-based tool for dynamic functional outcome after acute ischemic stroke and comparison with existing models
Source: BMC Neurol. 2014 Nov 25;14:214. doi: 10.1186/s12883-014-0214-z (PMC4255632; doi:10.1186/s12883-014-0214-z)
Supplement: Additional 1: Table S1. — Patient characteristics; Table S2. Discrimination of the DFS-AIS and 8 existing models for functional outcome after AIS in the validation cohort (n = 4,811); Table S3. Discrimination of the DFS-AIS and 8 existing models for mortality after AIS in the validation cohort (n = 4,811); Table S4. Discrimination of the DFS-AIS and 8 existing models for functional outcome after AIS in the overall cohort (n = 12,026); Table S5. showed comparison of discrimination of the DFS-AIS and 8 existing models for functional outcome at discharge, 3-month, 6-month and 1-year after AIS in the overall cohort. Table S6. showed comparison of discrimination of the DFS-AIS and 8 existing models for mortality at discharge, 3-month, 6-month and 1-year after AIS in the overall cohort. Figure S1. Patient flowchart; Figure S2. Discrimination of the DFS-AIS for dynamic functional outcome after AIS. Figure S3. Plot of observed versus predicted likelihood of good functional outcome at multiple time points after AIS; Additional file 1: The CNSR investigators; Additional file 1: Institutional Review Board within the CNSR network. [file 12883_2014_214_MOESM1_ESM.docx]

**Supplementary materials**

**Additional file 1: Table S1. Patient characteristics**

|  | **Patients included in the study**  **(N=12026)** | **Patients excluded for receiving thrombolysis***  **(N=389)** | **P value** |
| --- | --- | --- | --- |
| Demographics |  |  |  |
| Age, y, median (IQR) | 67 (57-75) | 64 (55-71) | <0.001 |
| Gender (male), n (%) | 7411 (61.6) | 247 (63.5) | 0.49 |
| Stroke risk factors, n (%) |  |  |  |
| Hypertension | 7703 (64.1) | 206 (53.0) | <0.001 |
| Diabetes mellitus | 2615 (21.7) | 62 (15.9) | 0.007 |
| Dyslipidemia | 1349 (11.2) | 41 (10.5) | 0.73 |
| Atrial fibrillation | 870 (7.2) | 48 (12.3) | <0.001 |
| Coronary artery disease | 1714 (14.3) | 78 (20.1) | 0.002 |
| History of stroke/TIA | 4113 (33.6) | 121 (31.1) | 0.21 |
| Current Smoking | 4750 (39.5) | 184 (47.3) | 0.002 |
| Excess alcohol consumption | 1844 (15.3) | 72 (18.5) | 0.10 |
| Pre-existing comorbidities, n (%) |  |  |  |
| Congestive heart failure | 239 (2.0) | 15 (3.9) | 0.02 |
| Valvular heart disease | 284 (2.4) | 16 (4.1) | 0.04 |
| COPD | 138 (1.1) | 6 (1.6) | 0.51 |
| Hepatic cirrhosis | 42 (0.4) | 0 (0.0) | 0.64 |
| Peptic ulcer or previous GIB | 411 (3.4) | 9 (2.3) | 0.32 |
| Alzheimer’s disease/Dementia | 166 (1.4) | 2 (0.5) | 0.18 |
| Cancer | 222 (1.8) | 4 (0.1) | 0.33 |
| Pre-stroke dependence (mRS≥3), n (%) | 1140 (9.5) | 25 (6.4) | 0.05 |
| Pre-admission antithrombotic therapy, n (%) | 2246 (18.7) | 68 (17.5) | 0.59 |
| Warfarin (for atrial fibrillation) | 272 (2.3) | 7 (1.8) | 0.72 |
| Antiplatelet using | 2208 (16.7) | 59 (15.2) | 0.45 |
| Pre-admission statins using, n (%) | 5175 (43.0) | 171 (44.0) | 0.72 |
| Transport to hospital by EMS, n (%) | 1826 (15.2) | 127 (32.6) | <0.001 |
| Time from onset to arrival (hours), median (IQR) | 24 (7-64) | 2.33 (1.00-11.1) | <0.001 |
| Admission SBP (mm Hg), median (IQR) | 150 (136-164) | 150 (130-165) | 0.52 |
| Admission DBP (mm Hg), median (IQR) | 89 (80-96) | 89 (80-100) | 0.74 |
| Admission NIHSS score, median (IQR) | 5 (2-9) | 10 (6.0-16.0) | <0.001 |
| OCSP subtypes, n (%) |  |  | <0.001 |
| Partial anterior circulation infarct (PACI) | 6698 (55.7) | 215 (55.3) |  |
| Total anterior circulation infarct (TACI) | 1035 (8.6) | 70 (18.0) |  |
| Lacunar infarction (LACI) | 2252 (18.7) | 44 (11.3) |  |
| Posterior circulation infarct (POCI) | 2009 (17.1) | 50 (15.4) |  |
| Admission blood glucose (mmol/L), median (IQR) | 6.2 (5.5-7.0) | 6.4 (5.7-7.6) | <0.001 |
| Length of hospital stay, median (IQR) | 14 (10-20) | 16 (11-22) | <0.001 |
| mRS≤2 within 1 year after onset, n (%) |  |  |  |
| At discharge | 8160 (67.9) | 208 (53.4) | <0.001 |
| At 3-month | 7994 (66.5) | 232 (59.6) | 0.005 |
| At 6-month | 8050 (66.9) | 236 (60.7) | 0.01 |
| At 12-mont | 8047 (66.9) | 240 (61.7) | 0.03 |
| Mortality within 1 year after onset, n (%) |  |  |  |
| At discharge | 468 (3.9) | 28 (7.2) | 0.001 |
| At 3-month | 990 (8.2) | 46 (11.8) | 0.01 |
| At 6-month | 1270 (10.6) | 54 (13.9) | 0.05 |
| At 12-mont | 1602 (13.2) | 62 (15.9) | 0.15 |

* Including both intravenous and intra-arterial thrombolysis;

Abbreviation: IQR, Interquartile Range; TIA, Transient Ischemic Attack; COPD, chronic obstructive pulmonary disease; mRS, modified Rankin Scale; EMS, Emergency Medical System; SBP, Systolic Blood Pressure; DBP, Diastolic Blood Pressure; NIHSS, National Institutes of Health Stroke Scale score; OCSP, Oxfordshire Community Stroke Project.

**Additional file 1: Table S2. Discrimination of the DFS-AIS and 8 existing models for functional outcome after AIS in the validation cohort (n=4,811)**

|  | **AUROC** | **95% C.I.** | **Δ AUROC*** | **P value^&^** | **Youden Index** | **Cutoff** | **Sensitivity** | **Specificity** | **PPV** | **NPV** |
| --- | --- | --- | --- | --- | --- | --- | --- | --- | --- | --- |
| **Discharge mRS≤2** |  |  |  |  |  |  |  |  |  |  |
| Weimar’s survival model (2004)^&^ | 0.797 | 0.785-0.808 | 0.04 | <0.0001 | 0.428 | … | 0.899 | 0.529 | 0.803 | 0.713 |
| Weimar’s functional model (2004)^#^ | 0.814 | 0.803-0.825 | 0.023 | <0.0001 | 0.458 | … | 0.910 | 0.548 | 0.811 | 0.743 |
| König’s survival model (2008)^¶^ | 0.784 | 0.772-0.796 | 0.053 | <0.0001 | 0.396 | … | 0.915 | 0.481 | 0.790 | 0.727 |
| König’s functional model (2008)^$^ | 0.809 | 0.798-0.820 | 0.028 | <0.0001 | 0.467 | … | 0.887 | 0.580 | 0.818 | 0.707 |
| GWTG score (with NIHSS score) (2010) | 0.789 | 0.777-0.801 | 0.048 | <0.0001 | 0.398 | 47 | 0.912 | 0.486 | 0.791 | 0.722 |
| IScore (1-year model) (2011) | 0.720 | 0.707-0.733 | 0.117 | <0.0001 | 0.413 | 35 | 0.895 | 0.518 | 0.799 | 0.698 |
| PLAN score (2012) | 0.747 | 0.735-0.760 | 0.09 | <0.0001 | 0.314 | 12 | 0.887 | 0.427 | 0.768 | 0.640 |
| ASTRAL score (2012) | 0.806 | 0.794-0.817 | 0.031 | <0.0001 | 0.430 | 25 | 0.902 | 0.528 | 0.803 | 0.718 |
| DFS-AIS (2014) | 0.837 | 0.824-0.850 | Reference | … | 0.478 | … | 0.900 | 0.578 | 0.820 | 0.730 |
| **3-month mRS≤2** |  |  |  |  |  |  |  |  |  |  |
| Weimar’s survival model (2004)^&^ | 0.810 | 0.799-0.821 | 0.031 | <0.0001 | 0.446 | … | 0.910 | 0.536 | 0.799 | 0.746 |
| Weimar’s functional model (2004)^#^ | 0.816 | 0.805-0.827 | 0.025 | <0.0001 | 0.456 | … | 0.915 | 0.541 | 0.802 | 0.758 |
| König’s survival model (2008)^¶^ | 0.802 | 0.791-0.814 | 0.039 | <0.0001 | 0.42 | … | 0.927 | 0.493 | 0.788 | 0.770 |
| König’s functional model (2008)^$^ | 0.816 | 0.805-0.827 | 0.025 | <0.0001 | 0.471 | … | 0.898 | 0.573 | 0.811 | 0.736 |
| GWTG score (with NIHSS score) (2010) | 0.787 | 0.775-0.798 | 0.054 | <0.0001 | 0.415 | 47 | 0.922 | 0.493 | 0.787 | 0.758 |
| IScore (1-year model) (2011) | 0.716 | 0.703-0.729 | 0.125 | <0.0001 | 0.395 | 35 | 0.893 | 0.502 | 0.785 | 0.699 |
| PLAN score (2012) | 0.761 | 0.749-0.773 | 0.08 | <0.0001 | 0.351 | 12 | 0.902 | 0.449 | 0.769 | 0.694 |
| ASTRAL score (2012) | 0.813 | 0.802-0.824 | 0.028 | <0.0001 | 0.438 | 25 | 0.909 | 0.529 | 0.797 | 0.743 |
| DFS-AIS (2014) | 0.841 | 0.828-0.853 | Reference | … | 0.466 | … | 0.911 | 0.555 | 0.806 | 0.754 |
| **6-month mRS≤2** |  |  |  |  |  |  |  |  |  |  |
| Weimar’s survival model (2004)^&^ | 0.813 | 0.802-0.824 | 0.028 | <0.0001 | 0.421 | … | 0.922 | 0.499 | 0.792 | 0.756 |
| Weimar’s functional model (2004)^#^ | 0.812 | 0.800-0.823 | 0.029 | <0.0001 | 0.447 | … | 0.910 | 0.537 | 0.803 | 0.743 |
| König’s survival model (2008)^¶^ | 0.809 | 0.797-0.820 | 0.032 | <0.0001 | 0.412 | … | 0.922 | 0.490 | 0.789 | 0.754 |
| König’s functional model (2008)^$^ | 0.815 | 0.803-0.826 | 0.026 | <0.0001 | 0.456 | … | 0.893 | 0.563 | 0.811 | 0.719 |
| GWTG score (with NIHSS score) (2010) | 0.776 | 0.764-0.788 | 0.065 | <0.0001 | 0.414 | 45 | 0.899 | 0.515 | 0.794 | 0.713 |
| IScore (1-year model) (2011) | 0.704 | 0.691-0.717 | 0.137 | <0.0001 | 0.377 | 35 | 0.885 | 0.492 | 0.783 | 0.675 |
| PLAN score (2012) | 0.764 | 0.752-0.776 | 0.077 | <0.0001 | 0.358 | 12 | 0.903 | 0.455 | 0.775 | 0.695 |
| ASTRAL score (2012) | 0.812 | 0.801-0.823 | 0.029 | <0.0001 | 0.43 | 25 | 0.905 | 0.525 | 0.798 | 0.728 |
| DFS-AIS (2014) | 0.841 | 0.828-0.854 | Reference | … | 0.451 | … | 0.907 | 0.544 | 0.805 | 0.739 |
| **1-year mRS≤2** |  |  |  |  |  |  |  |  |  |  |
| Weimar’s survival model (2004)^&^ | 0.808 | 0.796-0.819 | 0.033 | <0.0001 | 0.433 | … | 0.927 | 0.506 | 0.793 | 0.773 |
| Weimar’s functional model (2004)^#^ | 0.803 | 0.792-0.814 | 0.038 | <0.0001 | 0.445 | … | 0.914 | 0.531 | 0.800 | 0.754 |
| König’s survival model (2008)^¶^ | 0.804 | 0.793-0.815 | 0.037 | <0.0001 | 0.42 | … | 0.926 | 0.494 | 0.789 | 0.768 |
| König’s functional model (2008)^$^ | 0.807 | 0.796-0.818 | 0.034 | <0.0001 | 0.437 | … | 0.923 | 0.514 | 0.796 | 0.768 |
| GWTG score (with NIHSS score) (2010) | 0.766 | 0.754-0.778 | 0.075 | <0.0001 | 0.391 | 47 | 0.913 | 0.478 | 0.782 | 0.731 |
| IScore (1-year model) (2011) | 0.703 | 0.690-0.716 | 0.138 | <0.0001 | 0.37 | 35 | 0.884 | 0.486 | 0.779 | 0.673 |
| PLAN score (2012) | 0.766 | 0.753-0.778 | 0.075 | <0.0001 | 0.357 | 12 | 0.904 | 0.453 | 0.772 | 0.698 |
| ASTRAL score (2012) | 0.804 | 0.793-0.815 | 0.037 | <0.0001 | 0.426 | 25 | 0.905 | 0.521 | 0.795 | 0.729 |
| DFS-AIS (2014) | 0.840 | 0.829-0.850 | Reference | … | 0.461 | … | 0.912 | 0.549 | 0.805 | 0.752 |

*Δ AUROC denotes the difference in AUROC between the DFS-AIS and compared scores with regard to good functional outcome (mRS≤2) at different time points after AIS.

^&^ P value of comparing pairwise AUROC with Delong’s method.

^&^Originally developed for survival at 100 days after onset of acute cerebral ischemia;

^#^Originally developed for functional recovery at 100 days after onset of acute cerebral ischemia;

^¶^Originally developed for survival within 3 months after acute stroke;

^$^Originally developed for functional independence within 3 months after acute stroke;

Abbreviation；AIS, Acute Ischemic Stroke; AUROC, Area Under the Receiver Operating Characteristic Curve; C.I., Confidence Interval; PPV, Positive Predictive Value; NPV, Negative Predictive Value.

**Additional file 1: Table S3. Discrimination of the DFS-AIS and 8 existing models for mortality after AIS in the validation cohort (n=4,811)**

|  | **AUROC** | **95% C.I.** | **Δ AUROC*** | **P value^&^** | **Youden Index** | **Cutoff** | **Sensitivity** | **Specificity** | **PPV** | **NPV** |
| --- | --- | --- | --- | --- | --- | --- | --- | --- | --- | --- |
| **Mortality at discharge** |  |  |  |  |  |  |  |  |  |  |
| Weimar’s survival model (2004)^&^ | 0.778 | 0.766-0.789 | 0.023 | <0.0001 | 0.437 | … | 0.635 | 0.802 | 0.118 | 0.981 |
| Weimar’s functional model (2004)^#^ | 0.767 | 0.755-0.779 | 0.034 | <0.0001 | 0.451 | … | 0.656 | 0.795 | 0.118 | 0.982 |
| König’s survival model (2008)^¶^ | 0.776 | 0.764-0.787 | 0.025 | <0.0001 | 0.441 | … | 0.635 | 0.806 | 0.120 | 0.982 |
| König’s functional model (2008)^$^ | 0.772 | 0.760-0.784 | 0.029 | <0.0001 | 0.448 | … | 0.645 | 0.803 | 0.120 | 0.982 |
| GWTG score (with NIHSS score) (2010) | 0.757 | 0.745-0.769 | 0.044 | <0.0001 | 0.45 | 53 | 0.604 | 0.846 | 0.141 | 0.981 |
| IScore (1-year model) (2011) | 0.722 | 0.709-0.734 | 0.079 | <0.0001 | 0.395 | 50 | 0.520 | 0.875 | 0.148 | 0.978 |
| PLAN score (2012) | 0.738 | 0.725-0.750 | 0.063 | <0.0001 | 0.39 | 13 | 0.515 | 0.875 | 0.147 | 0.978 |
| ASTRAL score (2012) | 0.785 | 0.773-0.796 | 0.016 | <0.0001 | 0.427 | 25 | 0.645 | 0.782 | 0.110 | 0.982 |
| DFS-AIS (2014) | 0.801 | 0.774-0.828 | Reference | … | 0.521 | … | 0.647 | 0.874 | 0.232 | 0.977 |
| **Mortality at 3-month after onset** |  |  |  |  |  |  |  |  |  |  |
| Weimar’s survival model (2004)^&^ | 0.783 | 0.771-0.794 | 0.022 | <0.0001 | 0.451 | … | 0.653 | 0.798 | 0.220 | 0.964 |
| Weimar’s functional model (2004)^#^ | 0.777 | 0.765-0.789 | 0.028 | <0.0001 | 0.463 | … | 0.658 | 0.805 | 0.227 | 0.905 |
| König’s survival model (2008)^¶^ | 0.782 | 0.770-0.793 | 0.023 | <0.0001 | 0.451 | … | 0.627 | 0.824 | 0.237 | 0.962 |
| König’s functional model (2008)^$^ | 0.781 | 0.769-0.793 | 0.024 | <0.0001 | 0.455 |  | 0.651 | 0.804 | 0.224 | 0.964 |
| GWTG score (with NIHSS score) (2010) | 0.760 | 0.748-0.772 | 0.045 | <0.0001 | 0.427 | 51 | 0.575 | 0.852 | 0.252 | 0.959 |
| IScore (1-year model) (2011) | 0.725 | 0.712-0.737 | 0.08 | <0.0001 | 0.385 | 40 | 0.565 | 0.820 | 0.220 | 0.956 |
| PLAN score (2012) | 0.754 | 0.742-0.766 | 0.051 | <0.0001 | 0.394 | 11 | 0.692 | 0.702 | 0.168 | 0.963 |
| ASTRAL score (2012) | 0.788 | 0.777-0.800 | 0.017 | <0.0001 | 0.44 | 25 | 0.640 | 0.800 | 0.218 | 0.962 |
| DFS-AIS (2014) | 0.805 | 0.793-0.816 | Reference | … | 0.519 | … | 0.697 | 0.822 | 0.312 | 0.959 |
| **Mortality at 6-month after onset** |  |  |  |  |  |  |  |  |  |  |
| Weimar’s survival model (2004)^&^ | 0.799 | 0.787-0.810 | 0.018 | <0.0001 | 0.472 | … | 0.661 | 0.811 | 0.287 | 0.954 |
| Weimar’s functional model (2004)^#^ | 0.792 | 0.780-0.803 | 0.025 | <0.0001 | 0.482 | … | 0.665 | 0.817 | 0.296 | 0.955 |
| König’s survival model (2008)^¶^ | 0.799 | 0.787-0.810 | 0.018 | <0.0001 | 0.469 | … | 0.633 | 0.836 | 0.308 | 0.952 |
| König’s functional model (2008)^$^ | 0.796 | 0.784-0.807 | 0.021 | <0.0001 | 0.476 | … | 0.659 | 0.817 | 0.294 | 0.954 |
| GWTG score (with NIHSS score) (2010) | 0.770 | 0.758-0.782 | 0.047 | <0.0001 | 0.443 | 44 | 0.647 | 0.796 | 0.267 | 0.952 |
| IScore (1-year model) (2011) | 0.731 | 0.718-0.744 | 0.086 | <0.0001 | 0.395 | 35 | 0.592 | 0.803 | 0.257 | 0.945 |
| PLAN score (2012) | 0.768 | 0.756-0.780 | 0.049 | <0.0001 | 0.419 | 12 | 0.590 | 0.829 | 0.285 | 0.946 |
| ASTRAL score (2012) | 0.801 | 0.790-0.812 | 0.016 | <0.0001 | 0.461 | 25 | 0.649 | 0.812 | 0.285 | 0.953 |
| DFS-AIS (2014) | 0.817 | 0.806-0.828 | Reference | … | 0.525 |  | 0.687 | 0.838 | 0.395 | 0.946 |
| **Mortality at 12-month after onset** |  |  |  |  |  |  |  |  |  |  |
| Weimar’s survival model (2004)^&^ | 0.805 | 0.793-0.816 | 0.009 | <0.0001 | 0.499 | … | 0.701 | 0.798 | 0.408 | 0.931 |
| Weimar’s functional model (2004)^#^ | 0.791 | 0.779-0.802 | 0.023 | <0.0001 | 0.475 | … | 0.647 | 0.828 | 0.352 | 0.942 |
| König’s survival model (2008)^¶^ | 0.798 | 0.786-0.809 | 0.016 | <0.0001 | 0.469 | … | 0.622 | 0.847 | 0.317 | 0.940 |
| König’s functional model (2008)^$^ | 0.795 | 0.783-0.806 | 0.019 | <0.0001 | 0.473 | … | 0.645 | 0.828 | 0.352 | 0.942 |
| GWTG score (with NIHSS score) (2010) | 0.769 | 0.757-0.781 | 0.045 | <0.0001 | 0.439 | 44 | 0.634 | 0.805 | 0.321 | 0.939 |
| IScore (1-year model) (2011) | 0.724 | 0.711-0.736 | 0.09 | <0.0001 | 0.389 | 35 | 0.578 | 0.811 | 0.307 | 0.930 |
| PLAN score (2012) | 0.767 | 0.755-0.779 | 0.047 | <0.0001 | 0.417 | 12 | 0.578 | 0.839 | 0.342 | 0.932 |
| ASTRAL score (2012) | 0.797 | 0.785-0.808 | 0.017 | <0.0001 | 0.454 | 25 | 0.632 | 0.822 | 0.340 | 0.939 |
| DFS-AIS (2014) | 0.814 | 0.802-0.825 | Reference | … | 0.504 | … | 0.735 | 0.769 | 0.386 | 0.936 |

*Δ AUROC denotes the difference in AUROC between the DFS-AIS and compared scores with regard to mortality at different time points after AIS.

^&^ P value of comparing pairwise AUROC with Delong’s method.

^&^Originally developed for survival at 100 days after onset of acute cerebral ischemia;

^#^Originally developed for functional recovery at 100 days after onset of acute cerebral ischemia;

^¶^Originally developed for survival within 3 months after acute stroke;

^$^Originally developed for functional independence within 3 months after acute stroke;

Abbreviation；AIS, Acute Ischemic Stroke; AUROC, Area Under the Receiver Operating Characteristic Curve; C.I., Confidence Interval; PPV, Positive Predictive Value; NPV, Negative Predictive Value.

**Additional file 1: Table S4.** **Discrimination of the DFS-AIS and 8 existing models for functional outcome after AIS in the overall cohort (n=12,026)**

|  | **AUROC** | **95% C.I.** | **Δ AUROC*** | **P value^&^** | **Youden Index** | **Cutoff** | **Sensitivity** | **Specificity** | **PPV** | **NPV** |
| --- | --- | --- | --- | --- | --- | --- | --- | --- | --- | --- |
| **Discharge mRS≤2** |  |  |  |  |  |  |  |  |  |  |
| Weimar’s survival model (2004)^&^ | 0.795 | 0.788-0.802 | 0.044 | <0.0001 | 0.458 | … | 0.839 | 0.619 | 0.834 | 0.603 |
| Weimar’s functional model (2004)^#^ | 0.810 | 0.808-0.822 | 0.029 | <0.0001 | 0.505 | … | 0.797 | 0.708 | 0.852 | 0.623 |
| König’s survival model (2008)^¶^ | 0.782 | 0.775-0.790 | 0.057 | <0.0001 | 0.432 | … | 0.799 | 0.633 | 0.821 | 0.598 |
| König’s functional model (2008)^$^ | 0.809 | 0.802-0.816 | 0.030 | <0.0001 | 0.487 | … | 0.787 | 0.700 | 0.847 | 0.609 |
| GWTG score (with NIHSS score) (2010) | 0.795 | 0.787-0.802 | 0.044 | <0.0001 | 0.465 | 38 | 0.797 | 0.668 | 0.835 | 0.609 |
| IScore (1-year model) (2011) | 0.731 | 0.722-0.738 | 0.108 | <0.0001 | 0.419 | 35 | 0.897 | 0.522 | 0.798 | 0.706 |
| PLAN score (2012) | 0.749 | 0.741-0.757 | 0.090 | <0.0001 | 0.354 | 11 | 0.784 | 0.570 | 0.797 | 0.560 |
| ASTRAL score (2012) | 0.802 | 0.794-0.809 | 0.037 | <0.0001 | 0.461 | 23 | 0.817 | 0.644 | 0.829 | 0.625 |
| DFS-AIS (2014) | 0.839 | 0.830-0.848 | Reference | … | 0.552 | … | 0.787 | 0.765 | 0.876 | 0.629 |
| **3-month mRS≤2** |  |  |  |  |  |  |  |  |  |  |
| Weimar’s survival model (2004)^&^ | 0.812 | 0.805-0.819 | 0.030 | <0.0001 | 0.479 | … | 0.805 | 0.674 | 0.847 | 0.587 |
| Weimar’s functional model (2004)^#^ | 0.817 | 0.810-0.824 | 0.025 | <0.0001 | 0.500 | … | 0.772 | 0.728 | 0.849 | 0.617 |
| König’s survival model (2008)^¶^ | 0.806 | 0.799-0.813 | 0.036 | <0.0001 | 0.469 | … | 0.817 | 0.652 | 0.823 | 0.642 |
| König’s functional model (2008)^$^ | 0.818 | 0.810-0.824 | 0.024 | <0.0001 | 0.494 | … | 0.781 | 0.713 | 0.844 | 0.622 |
| GWTG score (with NIHSS score) (2010) | 0.791 | 0.783-0.798 | 0.051 | <0.0001 | 0.448 | 36 | 0.769 | 0.679 | 0.826 | 0.598 |
| IScore (1-year model) (2011) | 0.721 | 0.713-0.729 | 0.121 | <0.0001 | 0.396 | 35 | 0.895 | 0.501 | 0.781 | 0.707 |
| PLAN score (2012) | 0.767 | 0.760-0.775 | 0.075 | <0.0001 | 0.4 | 11 | 0.801 | 0.599 | 0.799 | 0.604 |
| ASTRAL score (2012) | 0.815 | 0.807-0.821 | 0.027 | <0.0001 | 0.478 | 22 | 0.760 | 0.718 | 0.843 | 0.602 |
| DFS-AIS (2014) | 0.842 | 0.834-0.850 | Reference | … | 0.572 | … | 0.797 | 0.775 | 0.875 | 0.658 |
| **6-month mRS≤2** |  |  |  |  |  |  |  |  |  |  |
| Weimar’s survival model (2004)^&^ | 0.815 | 0.808-0.822 | 0.026 | <0.0001 | 0.482 | … | 0.804 | 0.678 | 0.835 | 0.631 |
| Weimar’s functional model (2004)^#^ | 0.815 | 0.808-0.822 | 0.026 | <0.0001 | 0.492 | … | 0.767 | 0.725 | 0.849 | 0.606 |
| König’s survival model (2008)^¶^ | 0.811 | 0.804-0.811 | 0.030 | <0.0001 | 0.467 | … | 0.814 | 0.653 | 0.826 | 0.634 |
| König’s functional model (2008)^$^ | 0.818 | 0.811-0.825 | 0.023 | <0.0001 | 0.491 | … | 0.778 | 0.713 | 0.846 | 0.613 |
| GWTG score (with NIHSS score) (2010) | 0.784 | 0.777-0.792 | 0.057 | <0.0001 | 0.434 | 36 | 0.762 | 0.672 | 0.825 | 0.583 |
| IScore (1-year model) (2011) | 0.713 | 0.705-0.722 | 0.128 | <0.0001 | 0.386 | 35 | 0.890 | 0.496 | 0.782 | 0.691 |
| PLAN score (2012) | 0.771 | 0.763-0.778 | 0.070 | <0.0001 | 0.412 | 11 | 0.803 | 0.609 | 0.806 | 0.605 |
| ASTRAL score (2012) | 0.814 | 0.807-0.821 | 0.027 | <0.0001 | 0.479 | 22 | 0.758 | 0.721 | 0.846 | 0.596 |
| DFS-AIS (2014) | 0.841 | 0.833-0.849 | Reference | … | 0.557 | … | 0.790 | 0.767 | 0.873 | 0.644 |
| **1-year mRS≤2** |  |  |  |  |  |  |  |  |  |  |
| Weimar’s survival model (2004)^&^ | 0.814 | 0.807-0.821 | 0.027 | <0.0001 | 0.479 | … | 0.803 | 0.676 | 0.834 | 0.630 |
| Weimar’s functional model (2004)^#^ | 0.810 | 0.803-0.817 | 0.031 | <0.0001 | 0.476 | … | 0.821 | 0.655 | 0.828 | 0.644 |
| König’s survival model (2008)^¶^ | 0.812 | 0.805-0.819 | 0.029 | <0.0001 | 0.474 | … | 0.816 | 0.658 | 0.829 | 0.640 |
| König’s functional model (2008)^$^ | 0.814 | 0.807-0.821 | 0.027 | <0.0001 | 0.483 | … | 0.775 | 0.708 | 0.843 | 0.609 |
| GWTG score (with NIHSS score) (2010) | 0.775 | 0.767-0.782 | 0.066 | <0.0001 | 0.421 | 36 | 0.758 | 0.663 | 0.820 | 0.576 |
| IScore (1-year model) (2011) | 0.705 | 0.696-0.713 | 0.136 | <0.0001 | 0.374 | 35 | 0.886 | 0.488 | 0.778 | 0.680 |
| PLAN score (2012) | 0.770 | 0.763-0.778 | 0.071 | <0.0001 | 0.417 | 11 | 0.805 | 0.612 | 0.807 | 0.608 |
| ASTRAL score (2012) | 0.810 | 0.803-0.817 | 0.031 | <0.0001 | 0.467 | 22 | 0.755 | 0.712 | 0.841 | 0.589 |
| DFS-AIS (2014) | 0.841 | 0.833-0.849 | Reference | … | 0.556 | … | 0.791 | 0.765 | 0.872 | 0.644 |

*Δ AUROC denotes the difference in AUROC between the DFS-AIS and compared scores with regard to good functional outcome (mRS≤2) at different time points after AIS.

^&^ P value of comparing pairwise AUROC with Delong’s method.

^&^Originally developed for survival at 100 days after onset of acute cerebral ischemia;

^#^Originally developed for functional recovery at 100 days after onset of acute cerebral ischemia;

^¶^Originally developed for survival within 3 months after acute stroke;

^$^Originally developed for functional independence within 3 months after acute stroke;

Abbreviation；AIS, Acute Ischemic Stroke; AUROC, Area Under the Receiver Operating Characteristic Curve; C.I., Confidence Interval; PPV, Positive Predictive Value; NPV, Negative Predictive Value.

**Additional file 1: Table S5. Discrimination of the DFS-AIS and 8 existing models for mortality after AIS in the overall cohort (n=12,026)**

|  | **AUROC** | **95% C.I.** | **Δ AUROC*** | **P value^&^** | **Youden Index** | **Cutoff** | **Sensitivity** | **Specificity** | **PPV** | **NPV** |
| --- | --- | --- | --- | --- | --- | --- | --- | --- | --- | --- |
| **Mortality at discharge** |  |  |  |  |  |  |  |  |  |  |
| Weimar’s survival model (2004)^&^ | 0.785 | 0.777-0.792 | 0.015 | <0.0001 | 0.470 | … | 0.607 | 0.863 | 0.152 | 0.982 |
| Weimar’s functional model (2004)^#^ | 0.776 | 0.769-0.784 | 0.024 | <0.0001 | 0.473 | … | 0.598 | 0.875 | 0.162 | 0.982 |
| König’s survival model (2008)^¶^ | 0.784 | 0.776-0.791 | 0.016 | <0.0001 | 0.465 | … | 0.605 | 0.860 | 0.149 | 0.982 |
| König’s functional model (2008)^$^ | 0.781 | 0.773-0.781 | 0.019 | <0.0001 | 0.477 | … | 0.598 | 0.879 | 0.166 | 0.982 |
| GWTG score (with NIHSS score) (2010) | 0.776 | 0.768-0.783 | 0.024 | <0.0001 | 0.465 | 57 | 0.617 | 0.863 | 0.154 | 0.982 |
| IScore (1-year model) (2011) | 0.751 | 0.743-0.758 | 0.049 | <0.0001 | 0.419 | 40 | 0.609 | 0.812 | 0.116 | 0.981 |
| PLAN score (2012) | 0.751 | 0.744-0.759 | 0.049 | <0.0001 | 0.354 | 12 | 0.628 | 0.799 | 0.113 | 0.982 |
| ASTRAL score (2012) | 0.790 | 0.783-0.797 | 0.010 | <0.0001 | 0.461 | 25 | 0.671 | 0.778 | 0.109 | 0.983 |
| DFS-AIS (2014) | 0.800 | 0.793-0.807 | Reference | … | 0.552 | … | 0.618 | 0.861 | 0.137 | 0.982 |
| **Mortality at 3-month after onset** |  |  |  |  |  |  |  |  |  |  |
| Weimar’s survival model (2004)^&^ | 0.797 | 0.790-0.805 | 0.011 | <0.0001 | 0.480 | … | 0.663 | 0.817 | 0.246 | 0.964 |
| Weimar’s functional model (2004)^#^ | 0.790 | 0.783-0.798 | 0.018 | <0.0001 | 0.481 | … | 0.639 | 0.842 | 0.266 | 0.963 |
| König’s survival model (2008)^¶^ | 0.798 | 0.790-0.805 | 0.010 | <0.0001 | 0.479 | … | 0.659 | 0.820 | 0.248 | 0.964 |
| König’s functional model (2008)^$^ | 0.795 | 0.787-0.802 | 0.013 | <0.0001 | 0.483 |  | 0.648 | 0.835 | 0.261 | 0.964 |
| GWTG score (with NIHSS score) (2010) | 0.776 | 0.776-0.783 | 0.032 | <0.0001 | 0.458 | 52 | 0.606 | 0.852 | 0.270 | 0.960 |
| IScore (1-year model) (2011) | 0.741 | 0.733-0.749 | 0.067 | <0.0001 | 0.413 | 35 | 0.617 | 0.796 | 0.214 | 0.959 |
| PLAN score (2012) | 0.772 | 0.764-0.779 | 0.036 | <0.0001 | 0.443 | 12 | 0.624 | 0.819 | 0.237 | 0.960 |
| ASTRAL score (2012) | 0.799 | 0.792-0.806 | 0.009 | 0.008 | 0.457 | 25 | 0.659 | 0.798 | 0.227 | 0.963 |
| DFS-AIS (2014) | 0.808 | 0.801-0.815 | Reference | … | 0.493 | … | 0.621 | 0.872 | 0.304 | 0.962 |
| **Mortality at 6-month after onset** |  |  |  |  |  |  |  |  |  |  |
| Weimar’s survival model (2004)^&^ | 0.800 | 0.795-0.811 | 0.014 | <0.0001 | 0.479 | … | 0.651 | 0.828 | 0.309 | 0.953 |
| Weimar’s functional model (2004)^#^ | 0.795 | 0.787-0.802 | 0.019 | <0.0001 | 0.479 | … | 0.650 | 0.829 | 0.309 | 0.953 |
| König’s survival model (2008)^¶^ | 0.806 | 0.799-0.813 | 0.008 | 0.006 | 0.477 | … | 0.646 | 0.831 | 0.311 | 0.952 |
| König’s functional model (2008)^$^ | 0.800 | 0.793-0.807 | 0.014 | <0.0001 | 0.481 | … | 0.635 | 0.846 | 0.328 | 0.952 |
| GWTG score (with NIHSS score) (2010) | 0.776 | 0.769-0.784 | 0.038 | <0.0001 | 0.450 | 46 | 0.636 | 0.814 | 0.288 | 0.950 |
| IScore (1-year model) (2011) | 0.735 | 0.727-0.743 | 0.079 | <0.0001 | 0.405 | 35 | 0.600 | 0.805 | 0.267 | 0.945 |
| PLAN score (2012) | 0.776 | 0.768-0.783 | 0.038 | <0.0001 | 0.445 | 12 | 0.616 | 0.829 | 0.300 | 0.948 |
| ASTRAL score (2012) | 0.804 | 0.797-0.811 | 0.010 | 0.005 | 0.459 | 24 | 0.689 | 0.770 | 0.262 | 0.955 |
| DFS-AIS (2014) | 0.814 | 0.807-0.821 | Reference | … | 0.489 |  | 0.665 | 0.824 | 0.309 | 0.954 |
| **Mortality at 12-month after onset** |  |  |  |  |  |  |  |  |  |  |
| Weimar’s survival model (2004)^&^ | 0.805 | 0.797-0.812 | 0.011 | <0.0001 | 0.471 | … | 0.631 | 0.840 | 0.378 | 0.930 |
| Weimar’s functional model (2004)^#^ | 0.794 | 0.787-0.801 | 0.022 | <0.0001 | 0.469 | … | 0.646 | 0.823 | 0.359 | 0.938 |
| König’s survival model (2008)^¶^ | 0.807 | 0.799-0.815 | 0.009 | <0.0001 | 0.472 | … | 0.702 | 0.770 | 0.320 | 0.944 |
| König’s functional model (2008)^$^ | 0.800 | 0.793-0.807 | 0.016 | <0.0001 | 0.471 | … | 0.644 | 0.827 | 0.364 | 0.938 |
| GWTG score (with NIHSS score) (2010) | 0.774 | 0.766-0.781 | 0.042 | <0.0001 | 0.436 | 45 | 0.623 | 0.813 | 0.339 | 0.934 |
| IScore (1-year model) (2011) | 0.728 | 0.720-0.736 | 0.088 | <0.0001 | 0.398 | 35 | 0.583 | 0.815 | 0.327 | 0.927 |
| PLAN score (2012) | 0.772 | 0.764-0.779 | 0.044 | <0.0001 | 0.434 | 12 | 0.594 | 0.840 | 0.364 | 0.931 |
| ASTRAL score (2012) | 0.804 | 0.796-0.811 | 0.012 | 0.0002 | 0.454 | 24 | 0.672 | 0.782 | 0.322 | 0.940 |
| DFS-AIS (2014) | 0.816 | 0.809-0.823 | Reference | … | 0.486 | … | 0.718 | 0.768 | 0.322 | 0.947 |

*Δ AUROC denotes the difference in AUROC between the DFS-AIS and compared scores with regard to mortality at different time points after AIS.

^&^ P value of comparing pairwise AUROC with Delong’s method.

^&^Originally developed for survival at 100 days after onset of acute cerebral ischemia;

^#^Originally developed for functional recovery at 100 days after onset of acute cerebral ischemia;

^¶^Originally developed for survival within 3 months after acute stroke;

^$^Originally developed for functional independence within 3 months after acute stroke;

Abbreviation；AIS, Acute Ischemic Stroke; AUROC, Area Under the Receiver Operating Characteristic Curve; C.I., Confidence Interval; PPV, Positive Predictive Value; NPV, Negative Predictive Value.

|  |
| --- |
| **Additional file 1: Figure S1. patient flowchart** |

| **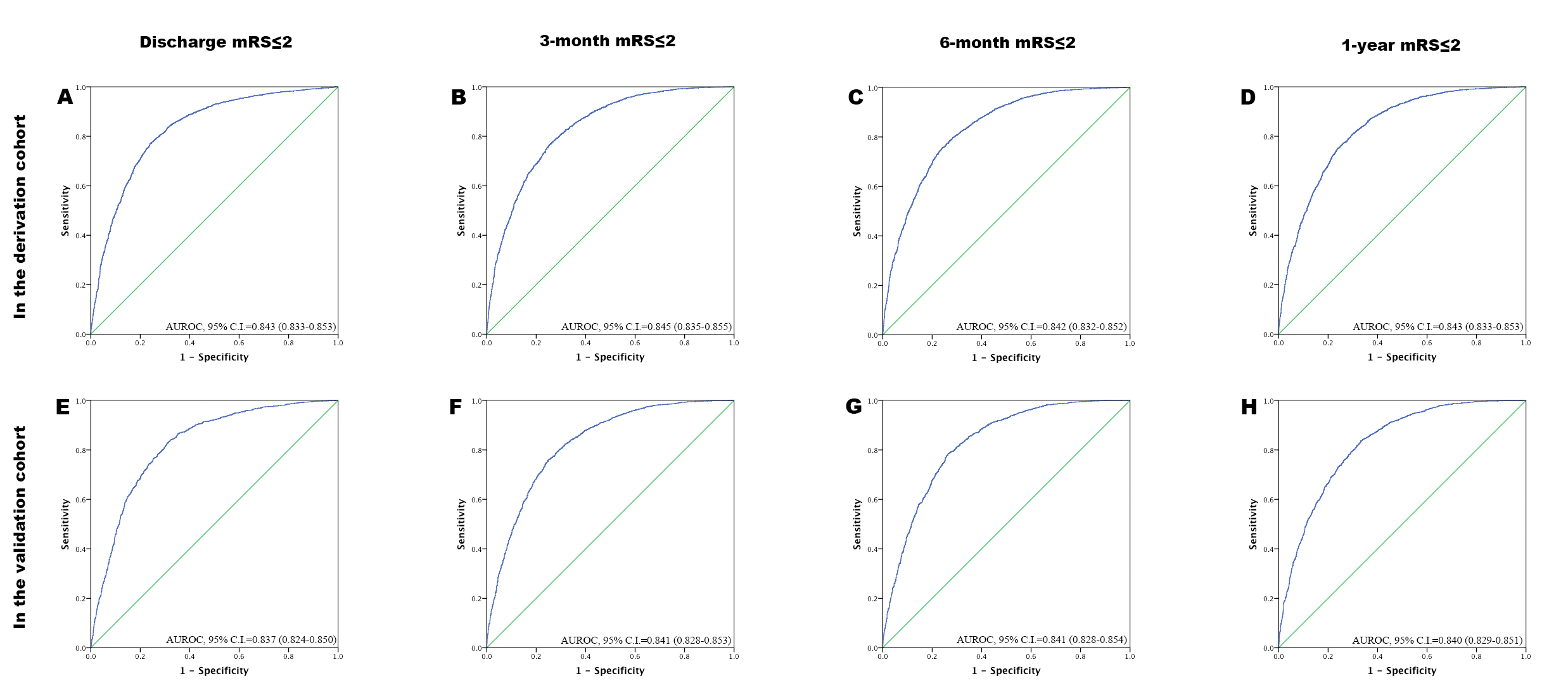** |
| --- |
| **Additional file 1: Figure S2:** Discrimination of the DFS-AIS for dynamic functional outcome after AIS |

**Legend:** The area under the receiver operating characteristic curve (AUROC) of the DFS-AIS for good functional outcome (mRS≤2) at discharge (A), 3-month (B), 6-month (C), and 1-year (D) after AIS in the derivation cohort was 0.843 (0.833-0.853), 0.845 (0.835-0.855), 0.842 (0.832-0.852), and 0.843 (0.833-0.853), respectively. Similar good discrimination was found in the validation cohort (AUROC range: 0.837-0.841) (E-H).

| **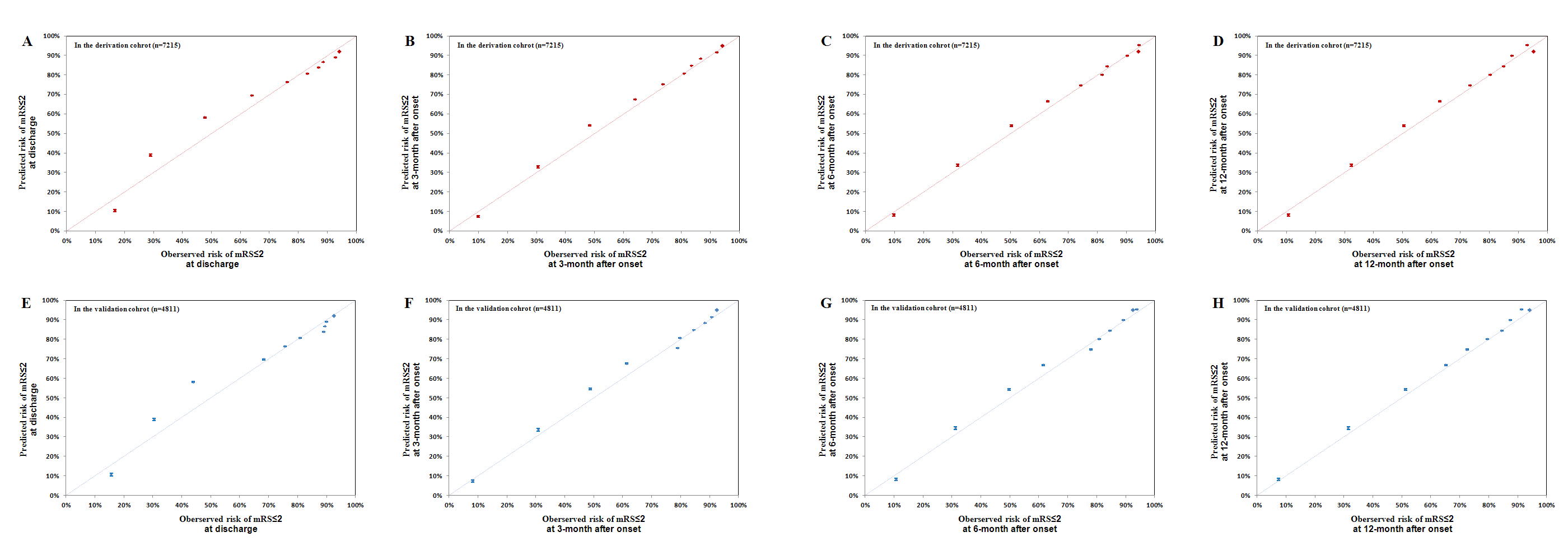** |
| --- |
| **Additional file 1: Figure S3:** Plot of observed versus predicted likelihood of good functional outcome at multiple time points after AIS |

**Legend:** Plot of observed versus predicted likelihood of good functional outcome (mRS≤2) at discharge, 3-month, 6-month, and 1-year after AIS with 95% confidence interval (C.I.) in the derivation and validation cohorts according to 10 deciles of predicted risk. Overall, there was a high correlation between observed and predicted likelihood of good functional outcome at discharge (A and E), 3-month (B and F), 6-month (C and G), and 1-year (D and H) after AIS in the derivation cohort (n=7,215; all r=0.99, P<0.001) and validation cohort (n=4,811; all r=0.99, P<0.001), which indicated excellent calibration.

**Additional file 1: Appendix A The CNSR investigators**

Yongjun Wang, Beijing Tiantan Hospital; Qi Bi, Beijing Anzhen Hospital; Weiwei Zhang, Beijing Military District Gengral hospital of Chinese People’s Liberation Army; Liying Cui, Peking Union Medical College Hospital of Peking University; Yuheng Sun, Beijing Jishuitan Hospital; Maolin He, Beijing Shijitan Hospital; Dongsheng Fan, Peking University Third Hospital; Xunming Ji, Beijing Xuanwu Hospital; Jimei Li, Beijing Friendship Hospital Affiliated to Capital Medical University; Fang Zhang, Beijing Guangwai Hospital; Kai Feng, Beijing Shunyi District Hospital; Xiaojun Zhang, Beijing Tongren Hospital; Yansheng Li, Shanghai Renji Hospital; Shaoshi Wang, Shanghai First Municipal People’s Branch hospital; Wei Fan, Zhongshan Hospital of Fudan University; Zhenguo Liu, Xin Hua Hospital Affiliated to Shanghai Jiao Tong University; Xiaojiang Sun, The sixth People’sHospital Affiliated to Shanghai JiaoTong University; Wei Li, Shanghai Ninth People’s Hospital Affiliated to Shanghai JiaoTong University; Jianrong Liu, ShanghaiRuijin Hospital; Xu Chen, Shanghai 8th People’s Hospital; Qingke Bai, Pudong New Area People’s Hospital; Dexiang Gu, Shanghai Yangpu Area Shidong Hospital; Xin Li, Shanghai Yangpu Area Center Hospital; Qiang Dong, Huashan Hospital of Fudan University; Yan Cheng, Tianjin Medical University Gengeal Hospital; Lan Yu, Tianjin Huanhu Hospital; Bin Li, Dagang Oilfield Gengeal Hospital; Tongyu Wang, Bohai Oilfield Hospital; Kun Zhao, Baodi District People’s Hospital of Tianjin; Chaodong Zhang, The First Affiliated Hospital of China Medical University; Dingbo Tao, The First Afflicated Hospital of Dlian Medical University; Lin Yin, The Second Affiliated Hospital of Dlian Medical University; Fang Qu, Dlian Second People’s hospital; Jingbo Zhang, Dlian Third People’s hospital; Jianfeng Wang, Dalian Central hospital; Ying Lian, Dalian Economic and Technological Development District Hospital; Fang Qu, Shenying Military District General hospital of Chinese People’s Liberation Army; Jun Fan, Shenyang Military District 202 Hospital; Ying Gao, National Traditional Chinese Medicine (TCM)Thrombus Treatment Center of Liaoning Province; Mingdong Cheng, En’liang hopital of Tai’an County; Jiang Wu, The First Clinical College of Jilin University; Huashan Sun, Jilin Chemical Industrial Group General hopital; Jinying Li, Jilin Oilfield General Hospital; Guozhong Li, The First Clinical College of Harbin Medical University; Yulan Zhu, The Second Clinical College of Harbin Medical University; Zichao Yang, The Fourth Clinical College of Harbin Medical University; Fengmin Yang, Daqing Oilfield General Hospital; Jun Zhou, Mudan Jiang Second hospital of Hailongjiang Province; Minxia Guo, Shaanxi Provincial People’s Hospital; Zhengyi Li, The First Afflicated Hospital of Medical College of Xian Jiaotong University; Qilin Ma, The First Hospital of Xiamen; Renbin Huang, Chenzhou First People’s Hospital; Bo Xiao, Xiangya Hospital of Centre-south University; Kangning Chen, Southwest Hospital; Xinyue Qin, The First Affiliated Hospital of Chongqing Medical University; Changlin Hu, The Second Affiliated Hospital of Chongqing Medical University; Li Gao, Chengdu Third Municipal People’s Hospital; Jinsheng Zeng, The First Affiliated Hospital of Sun Yat-Sen University; Anding Xu, The First Affiliated Hospital of Jinan University; Xiong Zhang, Guangdong People’s Hospital; Ming Shao, The First Affiliated Hospital of Guangzhou Medical University; Feng Qi, LiWan Hospital of GuangZhou Medical College; Weimin Xiao, Dungun Municipal People’s Hospital; Suping Zhang, Guangzhou Red Cross Hospital; Xiaoping Pan, Guangzhou First TMUNICIPAL People’s Hospital; Suyue Pan, Nan Fang Hospital; Yefeng Cai, Guangdong Provincial Hospital of Traditional Chinese Medicine; Qi Wan, Jiang Su People’s Hospital; Yun Xu, Drum Tower Hospital Affiliated to Nanjing Medical University Upper First-class Hospital; KaiFu Ke, he Affiliated Hospital of Nantong University Upper First class Hospital; Yuenan Kong,Wuxi Second People’s Hospital Upper First-class Hospital; Qing Di, Neurology Hospital Affiliated to Nanjing Medical University Upper First-class Hospital; Fengyang Shao, Jiangsu Province Lianyungang Hospital of TCM Upper First-class Hospital; Yajun Jiang, Jiangsu Province Hospital of TCM Upper First-class Hospital; Daming Wang, The First People’s Hospital of Changzhou Upper First-class Hospital; Li Guo, The Second Hospital of Hebei Medical University; Wencui Xue, Qinhuangdao C Hospital.

**Additional file 1: Appendix B Institutional review board within the CNSR network**

Institutional review board at Beijing Tiantan Hospital; Institutional review board at Beijing Anzhen Hospital; Institutional review board at Beijing Military District Gengral hospital of Chinese People’s Liberation Army; Institutional review board at Peking Union Medical College Hospital of Peking University; Institutional review board at Beijing Jishuitan Hospital; Institutional review board at Beijing Shijitan Hospital; Institutional review board at Peking University Third Hospital; Institutional review board at Beijing Xuanwu Hospital; Institutional review board at Beijing Friendship Hospital Affiliated to Capital Medical University; Institutional review board at Beijing Guangwai Hospital; Institutional review board at Beijing Shunyi District Hospital; Institutional review board at Beijing Tongren Hospital; Institutional review board at Shanghai Renji Hospital; Institutional review board at Shanghai First Municipal People’s Branch hospital; Institutional review board at Zhongshan Hospital of Fudan University; Institutional review board at Xin Hua Hospital Affiliated to Shanghai Jiao Tong University; Institutional review board at the sixth People’sHospital Affiliated to Shanghai JiaoTong University; Institutional review board at Shanghai Ninth People’s Hospital Affiliated to Shanghai JiaoTong University; Institutional review board at ShanghaiRuijin Hospital; Institutional review board at Shanghai 8th People’s Hospital; Institutional review board at Pudong New Area People’s Hospital; Institutional review board at Shanghai Yangpu Area Shidong Hospital; Institutional review board at Shanghai Yangpu Area Center Hospital; Institutional review board at Huashan Hospital of Fudan University; Institutional review board at Tianjin Medical University Gengeal Hospital; Institutional review board at Tianjin Huanhu Hospital; Institutional review board at Dagang Oilfield Gengeal Hospital; Institutional review board at Bohai Oilfield Hospital; Institutional review board at Baodi District People’s Hospital of Tianjin; Institutional review board at The First Affiliated Hospital of China Medical University; Institutional review board at The First Afflicated Hospital of Dlian Medical University; Institutional review board at The Second Affiliated Hospital of Dlian Medical University; Institutional review board at Dlian Second People’s hospital; Institutional review board at Dlian Third People’s hospital; Institutional review board at Dalian Central hospital; Institutional review board at Dalian Economic and Technological Development District Hospital; Institutional review board at Shenying Military District General hospital of Chinese People’s Liberation Army; Institutional review board at Shenyang Military District 202 Hospital; Institutional review board at National Traditional Chinese Medicine (TCM) Thrombus Treatment Center of Liaoning Province; Institutional review board at En’liang hopital of Tai’an County; Institutional review board at The First Clinical College of Jilin University; Institutional review board at Jilin Chemical Industrial Group General hopital; Institutional review board at Jilin Oilfield General Hospital; Institutional review board at The First Clinical College of Harbin Medical University; Institutional review board at The Second Clinical College of Harbin Medical University; Institutional review board at The Fourth Clinical College of Harbin Medical University; Institutional review board at Daqing Oilfield General Hospital; Institutional review board at Mudan Jiang Second hospital of Hailongjiang Province; Institutional review board at Shaanxi Provincial People’s Hospital; Institutional review board at The First Afflicated Hospital of Medical College of Xian Jiaotong University; Institutional review board at The First Hospital of Xiamen; Institutional review board at Chenzhou First People’s Hospital; Institutional review board at Xiangya Hospital of Centre-south University; Institutional review board at Southwest Hospital; Institutional review board at The First Affiliated Hospital of Chongqing Medical University; Institutional review board at The Second Affiliated Hospital of Chongqing Medical University; Institutional review board at Chengdu Third Municipal People’s Hospital; Institutional review board at The First Affiliated Hospital of Sun Yat-Sen University; Institutional review board at The First Affiliated Hospital of Jinan University; Institutional review board at Guangdong People’s Hospital; Institutional review board at The First Affiliated Hospital of Guangzhou Medical University; Institutional review board at LiWan Hospital of GuangZhou Medical College; Institutional review board at Dungun Municipal People’s Hospital; Institutional review board at Guangzhou Red Cross Hospital; Institutional review board at Guangzhou First TMUNICIPAL People’s Hospital; Institutional review board at Nan Fang Hospital; Institutional review board at Guangdong Provincial Hospital of Traditional Chinese Medicine; Institutional review board at Jiang Su People’s Hospital; Institutional review board at Drum Tower Hospital Affiliated to Nanjing Medical University Upper First-class Hospital; Institutional review board at the Affiliated Hospital of Nantong University Upper First class Hospital; Institutional review board at Wuxi Second People’s Hospital Upper First-class Hospital; Institutional review board at Neurology Hospital Affiliated to Nanjing Medical University Upper First-class Hospital; Institutional review board at Jiangsu Province Lianyungang Hospital of TCM Upper First-class Hospital; Institutional review board at Jiangsu Province Hospital of TCM Upper First-class Hospital; Institutional review board at The First People’s Hospital of Changzhou Upper First-class Hospital; Institutional review board at The Second Hospital of Hebei Medical University; Institutional review board at Qinhuangdao C hospital.
